# Biostatic Immunities of the Jewish Race in Europe

**Published:** 1869-09

**Authors:** 


### Biostatic Immunities of the Jewish Race in Europe.

Mr. Legoyt terminates with the following conclusions, an elaborate paper, on the above subject, recently read before the Paris Statistical Society:

"The facts which are here collected, and which are nearly all derived from official sources, are almost unanimous in demonstrating that the Jewish race is distinguished from the other European races, in a biostatic point of view, by the following phenomena: 1. Its general fecundity is less. 2. So is it, at least as a general rule, with regard to its legitimate fecundity. 3. It is especially so with regard to its natural or illegitimate fecundity. 4. In an equal number of births there are fewer children born dead, which indicates that the Jewish woman passes through her period of gestation more favorably than the Christian woman. 5. But the most remarkable privilege of the Jews is, without contradiction, their relative low mortality, and that even when they are members of the lowest classes of society. This lesser mortality is not (and we cannot too much insist on this point) the natural consequence of a lesser fecundity, as, with an equal number of births, they count fewer deaths, and that by calculating on Halley's method—that is, in supposing the births equal to the death (taking place at the same ages)—it is found that they have a mean and probable life which is longer than that of the autocthonic races. It would not be correct to say that this difference in mortality is due to a large relative preponderance of adults, since in Prussia, which is the only country in which this portion of the population has been enumerated by age, there is found to be a greater number of children in it than in the Catholic and Evangelical population. 6. We have, moreover, seen that, as a consequence of this characteristic physical aptitude, the Jewish race becomes acclimatized everywhere, and propagates itself under every latitude. 7. Finally, we have shown that the Jews are possessed of a special aptitude enabling them to struggle against infected media, and protecting them against contagious diseases."

After discussing the various explanations of these immunities offered by different observers, M. Legoyt states that he believes the greater longevity of this race may be explained by the following considerations: 1. The Jews marry earlier than the Christians, and thus derive at an earlier age the advantages which statistics show are incident to the married state. Still, from their well-known prudence and circumspection, it is not to be supposed that they enter upon this until prepared to meet its exigencies. Among them hasty and rash marriages, which are alike hurtful to the health of parents and children, are rare. 2. The fecundity being less, they can pay much more attention to the preservation of their children. 3. By reason of the small number of illegitimate children they have, they escape the exceptional mortaliy which sweeps away such children. 4. The Jew does not pursue any calling which demands very hard labor. He is neither an agriculturist, a laborer, mechanic, sailor, nor miner. Before all things, he is the shopkeeper, merchant, banker, artist, *savant*, man of letters or public functionary. 5. The Mosaic law contains ordinances which, being purely hygienic, must exercise a favorable influence on the health—*e. g.*, the verification of the condition of slaughtered animals, the frequency of ablution, the practice of circumcision, and the separation of the husband until a week after menstruation, etc. 6. The strength of the family feeling among the Jews. It is only when it absolutely is impossible, and without distinction of rank, that a Jewish woman does not suckle her child. The children, too, are the objects of incessant and most vigilant care, which indeed is returned by the respect and solicitude which these manifest for their parents, especially when aged or infirm. This is probably one cause of the rarity of suicide among the Jews. 7. The sobriety of the Jews is incontestable. 8. Throughout the entire Jewish community a warm feeling of charity for the indigent and miserable prevails. 9. The religious Jew is remarkable for his great serenity of mind, and his deep-seated faith in Providence and the high destinies of his race. The constancy, the *perenuite* of the Jewish temperament, is well reflected in his religious faith, which has remained immovable for so many ages. 10. The morality of the Jews, as deduced from criminal statistics, seems to be real, and is only an indication of those regular habits of life which exercise so great an influence on the duration of life.—*Medical Times and Gazette, July* 10, 1869.

